# The Epidemiological Analysis of Pseudorabies Virus and Pathogenicity of the Variant Strain in Shandong Province

**DOI:** 10.3389/fvets.2022.806824

**Published:** 2022-03-02

**Authors:** Qinghai Ren, Hongwei Ren, Jinyuan Gu, Jin Wang, Luyao Jiang, Song Gao

**Affiliations:** ^1^Key Laboratory of Avian Bioproducts Development, Ministry of Agriculture and Rural Affairs, Yangzhou University, Yangzhou, China; ^2^Jiangsu Co-innovation Center for Prevention and Control of Important Animal Infectious Diseases and Zoonoses, Yangzhou University, Yangzhou, China; ^3^Institutes of Agricultural Science and Technology Development, Yangzhou University, Yangzhou, China; ^4^College of Veterinary Medicine, Yangzhou University, Yangzhou, China; ^5^Dutch State Mines (DSM) Vitamin Co., Ltd. (Shandong), Liaocheng, China

**Keywords:** pseudorabies virus, variant, epidemiological analysis, genetic evolution, pathogenicity

## Abstract

Pseudorabies (PR) is a disease that is seriously endangering the pig industry in China. To understand the current prevalence of pseudorabies virus (PRV) in Shandong Province, China, 19,292 serum samples were collected from 16 locations in Shandong from 2018 to 2020. The *gE* antibody was detected by enzyme-linked immunosorbent assay. Ninety-seven suspected cases of PRV infection were collected from sick pigs vaccinated with Bartha-K61 to isolate PRV. The results showed that the average positive rate of the PRV *gE* antibody decreased from 38.20% in 2018 to 18.12% in 2020, but there was a high positive rate in sows. The isolation rate of PRV was 13.40% (13/97), and four strains were purified through plaque assay (named PRV-SD1, PRV-SD2, PRV-SD3, and PRV-SD4). The homology and genetic evolution of four PRV strains based on *gE, gC, gI*, and *TK* genes were analyzed and showed that these four strains shared more than 99.0% nucleotide homology with the variant PRV XJ5 strain, and they clustered in the same sub-branch with the domestic variant PRV strains, including JS-2012 and XJ5. Furthermore, the pathogenicity of the isolated variant strain was assessed by intranasal infection of 16-week-old pigs with 1 mL PRV-SD1 strain. The results of the animal experiment demonstrated that the PRV-SD1–infected pigs exhibited obvious clinical symptoms as early as 2 days post inoculation (dpi), and all infected pigs died within 1 week. The severe hyperemia of meninges and swelling of lungs and tonsils were observed. Histopathology analysis showed the obvious lymphocytes necrosis of tonsils, interstitial pneumonia, and viral encephalitis. Many positive staining cells were observed in tonsils and brains through immunohistochemistry staining assay. Viral shedding in oropharyngeal and rectal swabs were detected at 2 dpi, reached a peak at 3 dpi, and then gradually decreased. The detection of viral loads in the tissues showed that tonsils had the highest virus titer, further proving it may be the target organ of variant PRV infection. In conclusion, variant PRV strains were still highly prevalent in Shandong Province, and they had a strong pathogenicity in pigs.

## Introduction

Pseudorabies, also known as Aujeszky's disease, is caused by the pseudorabies virus (PRV) and is characterized by anorexia, respiratory distress, and neurological disorders in pigs. This disease can be transmitted by saliva, nasal discharge, and airborne particles. It has been a major viral disease in pigs, resulting in great economic losses to the pig industry worldwide (1). Although pigs are the primary hosts and reservoirs of PRV, this virus also infects a wide range of other animals, including ruminants and rodents (2). Moreover, cases of PRV infection in humans have been sporadically reported in the past (3). Therefore, PRV may represent a potential threat to public health.

PRV belongs to the *Alphaherpesvirinae* subfamily within the family *Herpesvirida* and possesses a double-stranded DNA genome approximately 145 kb in length, containing at least 72 genes and coding more than 100 proteins (4). Among these, the viral envelope proteins glycoprotein B (*gB*), *gC*, and *gD* have been studied in depth and play key roles in virus entry, virulence, and immunity induction. In particular, *gE* is a major virulence factor of PRV and determines tropism for the central nervous system (5). The commercial *gE*-deleted vaccines are widely used in prevention and control of PRV at present, therefore, the detection of the *gE* antibody is often used to differentiate wild-type PRV-infected pigs from the vaccinated pigs, eradicating PRV circulation from pigs in many countries (6).

However, the emergence of the variant PRV was first reported in Bartha-K61-vaccinated pig farms in China in 2011 (7), and PRV variation may partly be due to vaccination immune pressure over time (8). Since late 2011, the outbreaks of variant PRV have emerged in many pig herds in more than 20 provinces of China (9), and Hu et al. (10) reported an outbreak of variant PRV in some pig farms in Shandong Province in 2013, which caused heavy economic losses. Although Shandong is a large pig-raising province, the efficiency of breeding is still not well-developed, and the prevalence of epidemic diseases is high. As PRV is recognized as one of the major infectious diseases in pigs, it is necessary to understand the prevalence and pathogenicity of variant PRV in Shandong Province. In the current study, a large number of serum samples and clinical cases in the province were collected for the prevalence investigation and isolation of PRV. Meanwhile, studies on the pathogenicity of variant PRV in pigs were performed. These results will provide data support for the prevalence of PRV in Shandong and the scientific basis for the prevention and control of PRV.

## Materials and Methods

### Samples and Cells

From 2018 to 2020, a total of 19,292 porcine serum samples were collected from 16 locations in Shandong Province, covering the entire province (Table 1). These serum samples were taken from sows, boars, replacement pigs, piglets, nursery pigs, and fattening pigs. Meanwhile, 97 suspected cases of PRV infection were collected from sick pigs at some routinely vaccinated farms. Lymph glands, lungs, tonsils, and brains were sampled for detection and isolation of PRV as well as for histopathological examination. Vero cells were cultured in Gibco Dulbecco's modified Eagle's medium (DMEM) supplemented with 10% fetal bovine serum (HyClone Labs, Logan, UT, USA), 100 U penicillin/ml, and 100 μg streptomycin/ml at 37°C in a 5% CO_2_ incubator.

**Table 1:** The specific sampling number in each area of Shandong in 2018-2020.

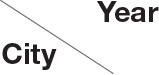	**2018**	**2019**	**2020**
Jinan	783	1170	945
Qingdao	842	910	692
Weifang	520	718	755
Linyi	65	131	251
Dongying	585	782	377
Yantai	263	713	560
Zibo	62	260	188
Jining	60	195	377
Taian	260	587	251
Weihai	195	127	188
Rizhao	450	128	314
Binzhou	1171	130	566
Dezhou	260	65	440
Liaocheng	915	459	388
Zaozhuang	64	65	0
Heze	0	65	0
Total	6495	6505	6292

### Enzyme-Linked Immunosorbent Assay (ELISA)

All serum samples were detected for *gE* antibody level to differentiate the wild strains from the vaccinated ones using the commercial ELISA kit (IDEXX Laboratories, Westbrook, ME, USA), according to the manufacturer's instructions. The results were determined by the formula S/N = OD560 test serum/OD650 negative control serum. S/N <0.6 was considered to be positive, 0.6 < S/N <0.7 was suspected, and S/N > 0.7 was negative.

### Detection, Isolation, and Plaque Assays of PRV

The mixed samples were homogenized in 1 ml of DMEM containing penicillin (100 U/mL) and streptomycin (100 μg/mL) and centrifuged at 3,000 rpm for 10 min. The supernatant was collected and used for DNA extraction according to the manufacturer's instructions for the TIANamp Genomic DNA Kit (TIANGEN, Beijing, China). Then, the extracted DNA was used for PRV detection through polymerase chain reaction (PCR) using the *gE* gene primer (10). The reaction condition of PCR was 94°C for 5 min, followed by 30 cycles of 94°C for 30 s, 58°C for 30 s, and 72°C for 50 s, with a final extension at 72°C for 10 min. The PCR products were subjected to electrophoresis on a 1% agarose gel and the target bands were visualized with an ultraviolet light transilluminator. The positive samples of PRV were selected for virus isolation. Specifically, the supernatants of positive homogenates were filtered using a 0.22 μm filter (Millipore, Shanghai, China) and inoculated into Vero cell monolayers. The cells were incubated at 37°C for 3 days and the cytopathic effect (CPE) was monitored. Then the supernatants of cultures showing CPE were harvested and confirmed by PCR. The isolated strains were stored at −80°C.

PRV strains were further purified by plaque assay. Briefly, Vero cells were seeded into 6-well plates and inoculated with a series of 10-fold dilutions (10^−1^ to 10^−6^) of PRV. An uninfected control was included in the experiment. The plates were incubated at 37°C for 1 h, after which the supernatants were removed, and DMEM containing 1% low-melting agarose was added to each well, and the plates were placed in an incubator to allow plaque formation. Plaques of a suitable size were selected and inoculated into 1 mL DMEM, frozen, and thawed three times. The virus stock was inoculated into Vero cells for propagation, and titers were calculated as median tissue culture infective dose (TCID_50_) by the Reed–Muench method (11).

### PCR Amplification

The full lengths of *gC, gE, gI*, and *TK* genes of PRV strains were amplified by PCR using the primers (Table 2). The PCR was conducted in a reaction volume of 20 μL, and the cycling conditions were 94°C for 5 min, 35 cycles of denaturation at 94°C for 1 min, annealing at 58–62°C for 1 min, with an extension at 72°C for 1 min, followed by a final extension at 72°C for 10 min. To validate the assay, the PCR products were subjected to electrophoresis on a 1% agarose gel, purified, and cloned into the pMD18-T vector, and the recombinant plasmids were sequenced for confirmation.

**Table 2:** Primer sequences designed for target genes.

**Genes name**	**Primer sequence**	**Annealing temperature**
*gC*	F: CCGTTTCCTGATTCACGC	61°C
	R: CGCAGATGATGTCCCAGC	
*gE*	F: CTCTGCGTGCTGTGCTCC	61.5°C
	R: CCTCGTCGCTGCTGAACT	
*gI*	F: GGGGTATCGCCTCCTGGG	62°C
	R: TCGGGACCTCGGTGACGG	
*TK*	F: TCACCGGGTGTCCATCTTCA	58°C
	R: TCCAGAAACAGCAGCGTCCC	

### Nucleotide Homology and Phylogenetic Tree Analyses

Seventeen PRV strains served as the reference strains (Table 3). The homologies of nucleic acid sequences between the reference strains and PRV strains isolated in our study were performed using MegAlign 7.1.0 software (DNASTAR, Madison, WI, USA). The phylogenetic analysis of PRV strains was made based on the full lengths of *gC, gE, gI*, and *TK* genes. The tree was constructed using the neighbor–joining method within MEGA 6.0 (12) with bootstrap analyses involving 1,000 replicates.

**Table 3:** The information of PRV reference strains for phylogenetic tree construction.

**Strains**	**GenBank accession number**	**Species**	**Year isolation**	**Origin**
Kaplan	JF797218	Swine	2011	Hungary
JS-2012	KP098534	Swine	2012	China
Ea	KU315430	Swine	1990	China
XJ5	Unpublished	Swine	2014	China
Namyangju	GQ325659.1	Swine	1987	South Korea
HNB	KM189914.3	Swine	2012	China
SC	KT809429.1	Swine	1986	China
LA	KU552118.1	Swine	1997	China
HLJ8	KT824771.1	Swine	2013	China
HN1201	KP722022.1	Swine	2012	China
HNX	KM189912.1	Swine	2012	China
TJ	KJ789182.1	Swine	2012	China
ZJ01	KM061380.1	Swine	2012	China
HeN1	KP098534.1	Swine	2012	China
Becker	JF797219.1	Swine	2011	USA
Ea(Hubei)	KX423960.1	Swine	1993	China
Fa	KM189913.1	Swine	2012	China

### Animal Experiment

Ten healthy growing pigs (Duroc × Landrace × Yorkshire hybrid) were bought from a pig farm without PRV vaccination and outbreaks of field strains in Liaocheng city, Shandong Province. PRV, porcine reproduction and respiratory syndrome virus, classical swine fever virus, and porcine circovirus two were tested by PCR methods as described (10), and all these viruses were negative. When the pigs were 16 weeks old, a PRV challenge experiment was conducted. First, the pigs were randomly divided into two groups (5 pigs/group) and raised separately. In group I, five pigs were inoculated intranasally with 1 mL PRV strain PRV-SD1 (1 × 10^6.7^ TCID_50_/0.1 mL). In group II, all pigs were injected with equal sterile PBS in the same manner, as a negative control group.

After the challenge, the pigs were observed continuously for 14 days and clinical symptoms, such as depression, anorexia, dyspnea, and ataxia were recorded. The rectal temperatures were measured. Oropharyngeal and rectal swabs of each pig were collected from 2 to 7 days post-inoculation (dpi) and were tested for viral shedding by the TCID_50_ method as described above. At 14 dpi, all pigs were euthanized with an overdose of pentobarbital by intracardial injection, and the gross lesions were observed. Meanwhile, the lymph glands, lungs, tonsils and brains were collected, and part of the tissues were fixed with 4% paraformaldehyde solution for histopathological examination. Other tissues were stored at −80°C for viral titers analyses using TCID_50_. During the experiment period, all pigs were housed in the isolators and had *ad libitum* access to feed and water.

### Histopathology Analysis and Immunohistochemistry Staining

The tissue samples (lymph gland, lung, tonsil and brain) were fixed with 4% paraformaldehyde and embedded in paraffin. The sections of the fixed tissues were cut to 3 μm. Some sections were stained with hematoxylin and eosin (H&E) and others were used for immunohistochemistry staining. H&E staining sections were observed by light microscopy for histopathological analysis.

The immunohistochemistry staining was performed as previous described (13), but the primary anti-PRV mouse monoclonal antibody used to detect PRV antigens was stored in our laboratory.

### Statistics Analysis

All data were expressed as means ± standard deviations (SD) and processed by GraphPad Prism 5.0 (GraphPad Software, San Diego, CA, USA). Student's *t*-test was used to analyze the statistical difference by SPSS 19.0 (IBM, Armonk, NY, USA). *P* < 0.05 was considered statistically significant.

## Results

### The Serological Investigation of Wild-Type PRV Circulated in Shandong Province

**Figure 1:**
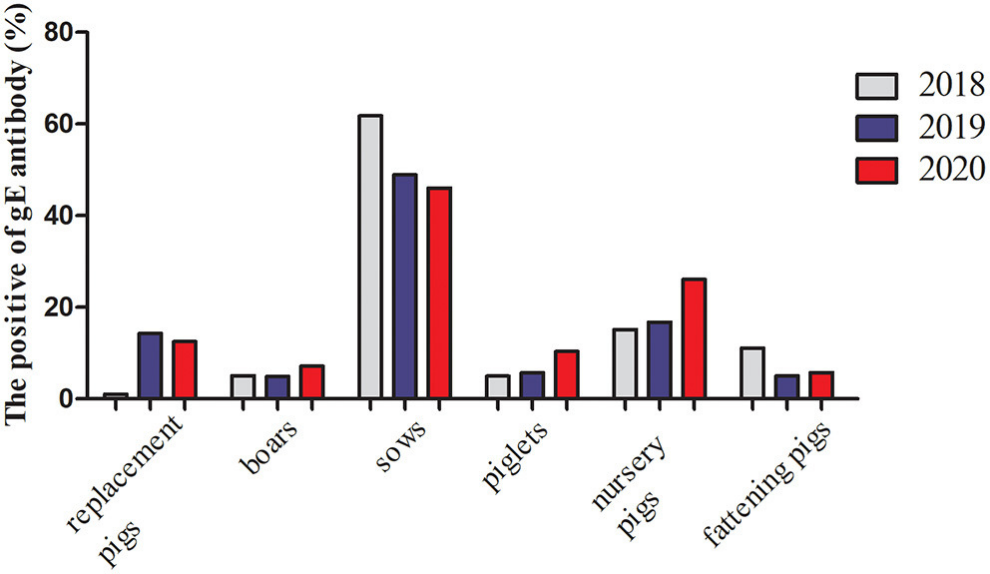
*gE* antibody positive rates of serums in the different pig herds in Shandong Province from 2018 to 2020.

From January 2018 to December 2020, 19,292 serum samples were collected from 16 locations in Shandong Province. Among these, 6,495 samples were collected in 2018, 6,505 in 2019, and 6,292 in 2020. According to the growth stage of the pigs, 7,909 serum samples were from sows, 1,352 were from boars, 1,736 from replacement pigs, and 3,473, 2,315, and 2,507 from nursery pigs, piglets, and fattening pigs, respectively. The detection results showed that the average positive rate of PRV *gE* antibody had decreased from 38.20% in 2018 to 18.12% in 2020, displaying a declining trend. However, the positive rates varied greatly in the different pig herds, with the positive rate of *gE* antibody in sows being about 46.0–61.8% in the past 3 years, which was significantly higher than those from other herds (Figure 1).

### The Isolation and Titer Determination of PRV Strains

In the current study, 97 suspected PRV cases were collected. Of these, 13 samples were found to be positive for the PRV *gE* gene using the PCR method, with the positive rate being 13.40% in the 3-year period. The PRV positive tissues were processed and inoculated into Vero cells for virus isolation. At 48 h post-inoculation, the cells showed obvious CPE, such as vacuoles, fragmentation, and necrosis. Furthermore, plaque assay was conducted for virus purification, and four PRV strains were obtained and named PRV-SD1, PRV-SD2, PRV-SD3, and PRV-SD4. Virus titers were further determined by TCID_50_, their titers being 10^6.7^ TCID_50_/0.1 mL, 10^6.9^ TCID_50_/0.1 mL, 10^6.2^ TCID_50_/0.1 mL, and 10^6.5^ TCID_50_/0.1 mL, respectively.

### The Homology and Phylogenetic Tree Analyses of PRV Strains

The glycoprotein genes *gC, gE*, and *gI*, and the virulence gene *TK* of four PRV strains were amplified for nucleotide acid homology analysis. The results showed that the sequence identities of these four genes among the strains obtained in this study ranged from 99.3 to 100%. Notably, the sequences of the *gC, gE, gI*, and *TK* genes of the isolated strains shared more than 99.1% identity with the PRV variant strain XJ5. Of these, the *gC* nucleotide sequence homologies of these four strains were more than 99.8% compared with the XJ5 strain, indicating that PRV-SD1, SD2, SD3, and SD4 were all variant strains.

**Figure 2:**
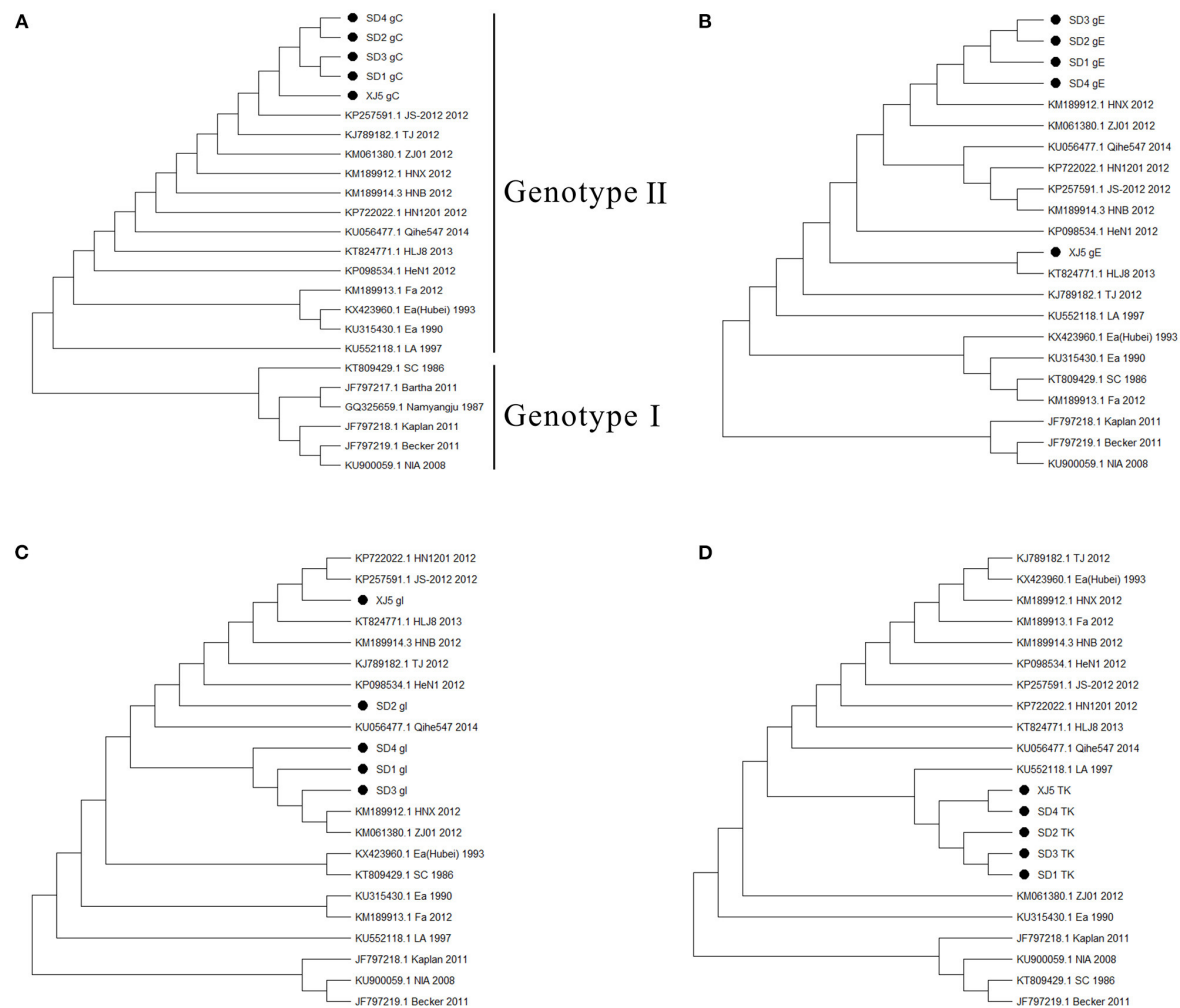
Phylogenetic trees based on *gC, gE, gI*, and *TK* genes of PRV strains. **(A)** *gC*, **(B)** *gE*, **(C)** *gI*, and **(D)** *TK*. The tree was constructed by the neighbor–joining method, with 1,000 bootstrap replicates using MEGA 6.0 software.

Phylogenetic trees based on the *gC, gE, gI*, and *TK* genes of the PRV strains were constructed. The results showed that the strains of genotypes I and II were all clearly separated from each other in the phylogenetic tree, and the isolated strains in our study belonged to genotype II (Figure 2A). Moreover, four gene sequences of the isolated strains were mainly clustered to an independent branch of the tree (Figures 2A–D). Overall, these four strains had a close genetic relationship with the variant PRV JS-2012 and XJ5 strains and were different from the Ea strain isolated earlier in China. However, they were also quite different from the Kaplan strain isolated from abroad, which belonged to genotype I.

### The Pathogenicity of the PRV Strain in Pigs

**Figure 3:**
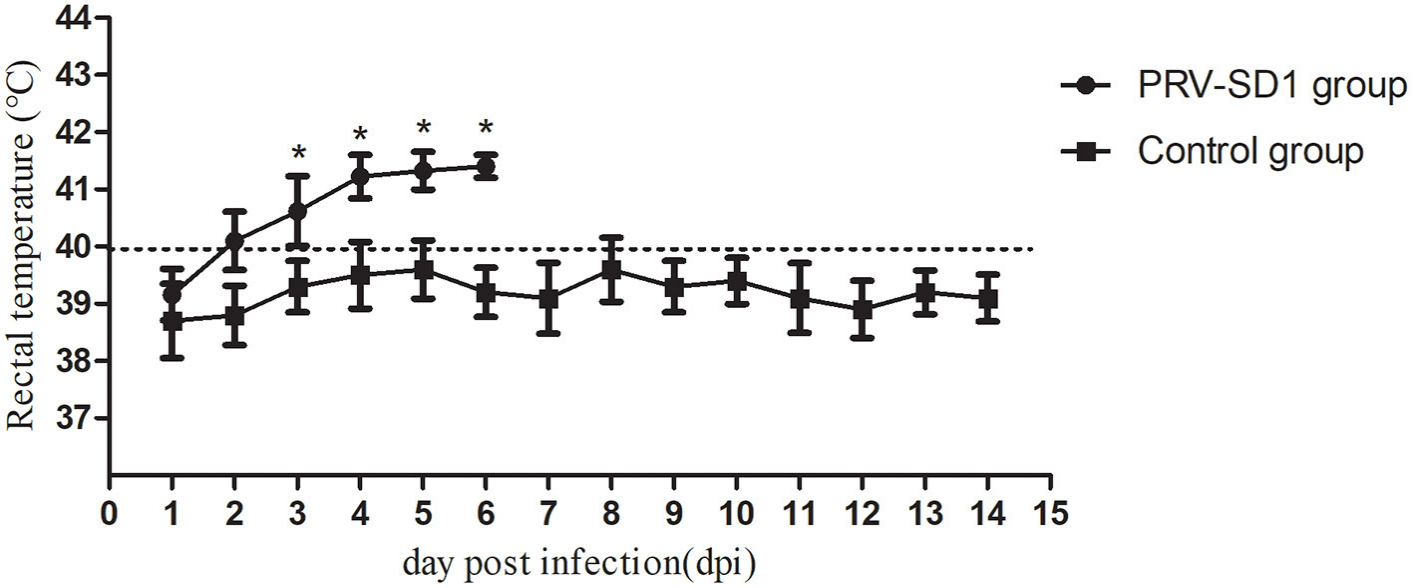
The dynamics of pigs' rectal temperature after virus challenge. Clinical fever was set at 40°C. *Indicates statistically significant difference (*P* < 0.05).

To analyze the pathogenicity of the variant PRV strain in pigs, animal experiments were carried out. The results showed that all the pigs in group I died within 1 week after the challenge, while the control pigs in group II survived and remained healthy throughout the experiment. Specifically, at 2 dpi, the PRV-infected pigs showed symptoms of shaking head and sneezing. At 3–4 dpi, the symptoms of depression, loss of appetite, frequent sneezing, and shaking were observed in all five infected pigs. The sick pigs showed dyspnea, one pig developed ataxia (in particular, excessive salivation), and then died at 5 dpi. Two pigs each died at 6 dpi and 7 dpi, with symptoms similar to the dead pig at 5 dpi. The PRV-infected pigs developed fever at 2 dpi and lasted for consecutive 5 days, and their body temperature was significantly higher than that of the control group (Figure 3, *P* < 0.05).

**Figure 4:**
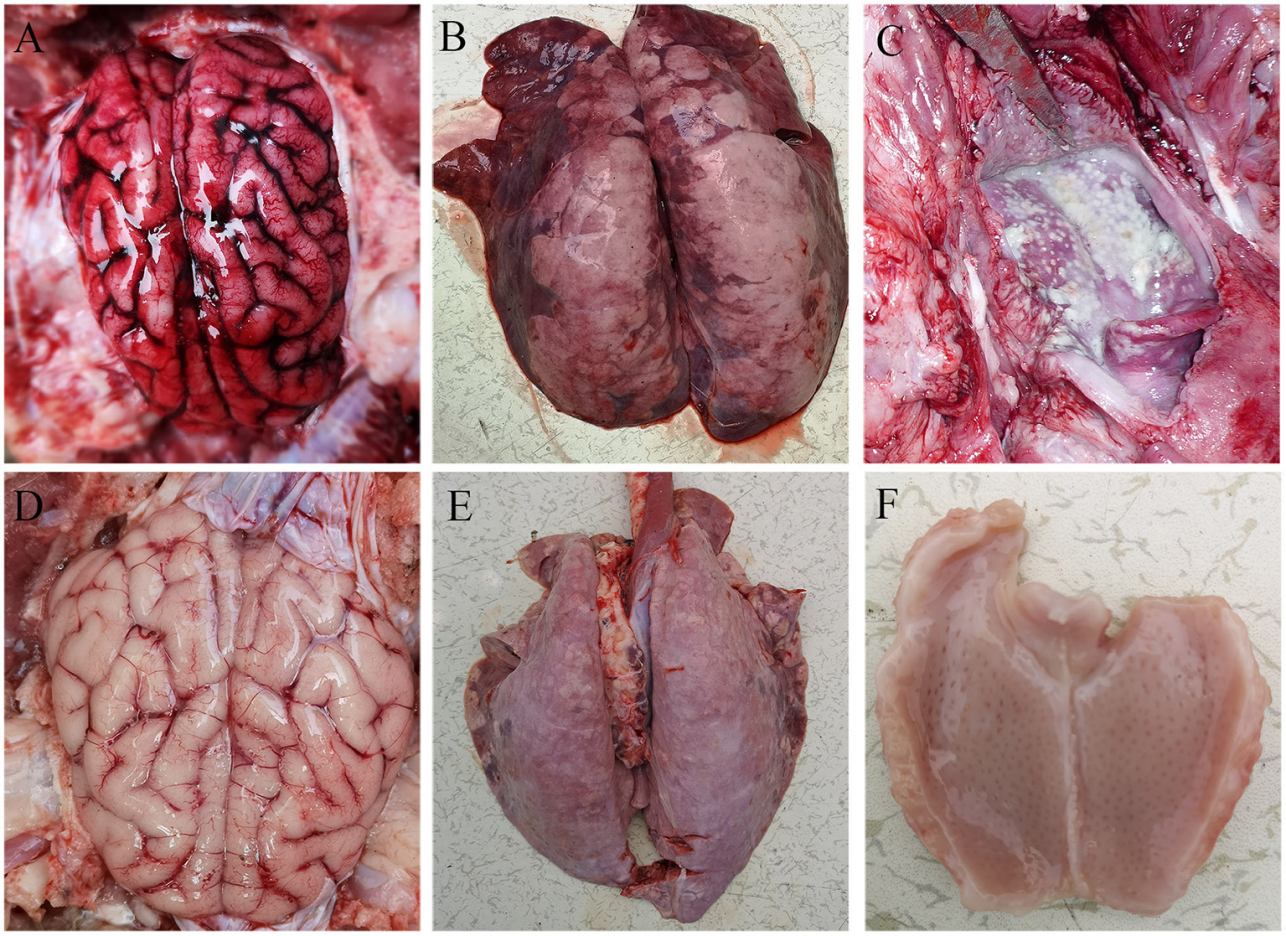
Representative gross lesions in pigs infected with variant PRV-SD1 strain. **(A)** The edema and hyperemia in the brain. **(B)** Pulmonary congestion and swelling. **(C)** Yellow-white exudate on the surface of the tonsil. **(D–F)** are the normal brain, lung and tonsil from the healthy pigs, respectively.

**Figure 5:**
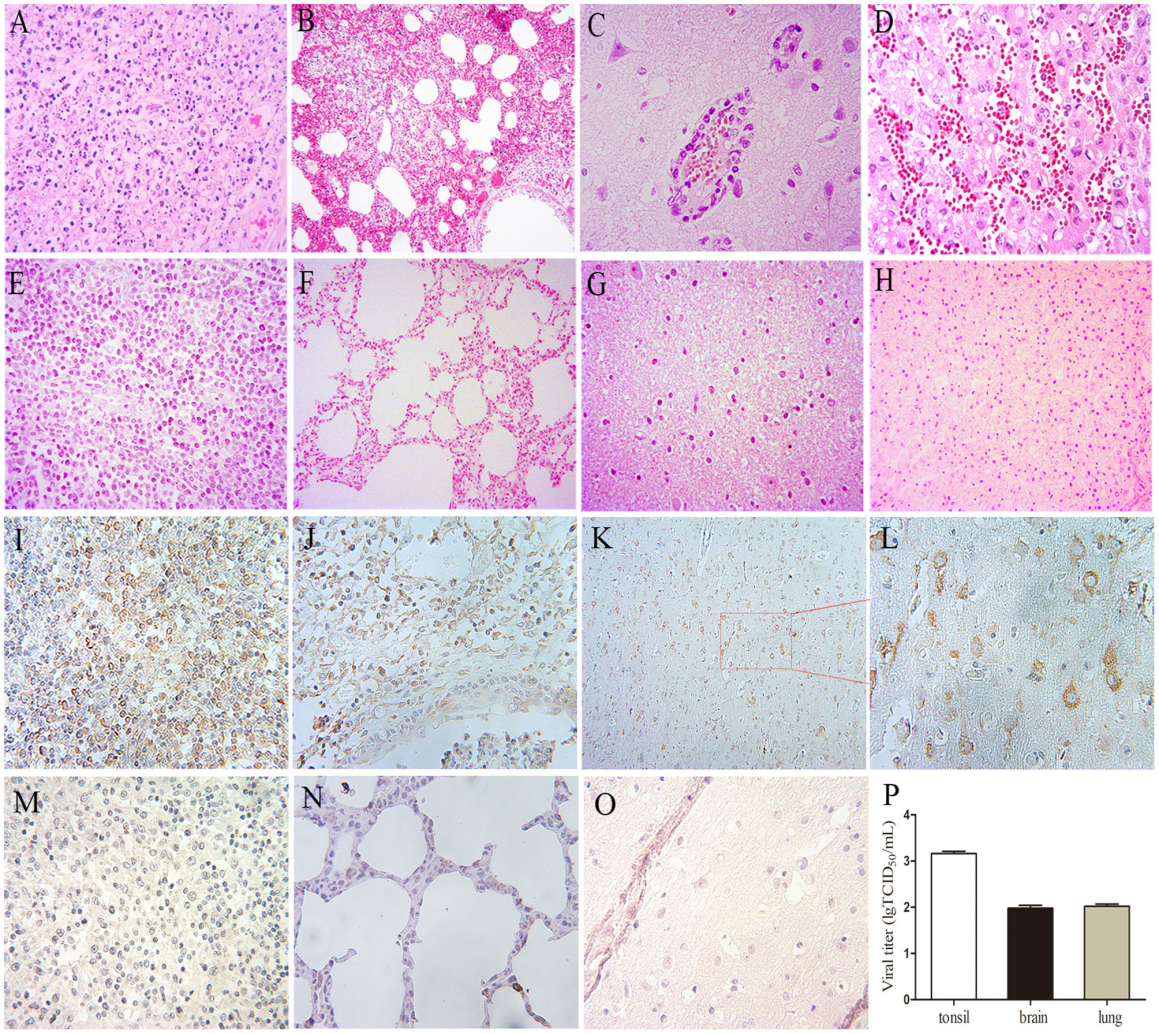
The results of H&E, immunohistochemistry staining, and viral loads in tissues in pigs. **(A**–**H)** H&E staining. **(A)** Lymphocyte necrosis of the tonsil, amplification 400×, **(B)** Serious hyperemia of vessels in alveolar wall, 200×, **(C)** Typical vascular cuff in the brain, 400×, **(D)** Hemorrhage of the liver, 400×. **(E–H)** are the healthy tonsil, lung, brain, and liver, respectively, from the control pigs. **(I–O)** Immunohistochemistry staining. **(I)** Tonsil, 400×, **(J)** Lung, 400×, **(K,L)** Brain, 200×, 400×, respectively. **(M–O)** Immunohistochemistry staining of corresponding tonsil, lung, and brain samples of control pigs. **(P)** Viral loads in the tonsil, lung, and brain of the PRV-infected pigs at 4 dpi, the data were expressed as means ± standard deviations (SD), error bars represent standard errors of the samples' means.

All pigs including the control group were subjected to autopsy at 14 dpi. Five pigs in the challenged group showed typical gross lesions, including cerebral edema, meningeal hyperemia (Figure 4A), severe lung congestion, and swelling (Figure 4B), and tonsil mucosa swelling, and bleeding, with a layer of yellow-white exudate on the surface (Figure 4C). In contrast, the tissues of the control pigs did not exhibit any obvious lesions (Figures 4D–F). Consistent with the above findings, H&E staining results showed that the dead pigs in the challenged group had numerous necroses of lymphocytes in tonsils (Figure 5A), extensive hyperemia of vessels in the alveolar wall (Figure 5B), lymphocyte infiltration around the small vessels in brains (Figure 5C), and hemorrhage, and degenerative necrosis of hepatocytes (Figure 5D). The tissues from the control pigs did not show any microscopic lesions (Figures 5E–H). To further determine the distribution of the PRV variant strain, the immunohistochemistry staining of tonsils, lungs, and brains of the infected pigs was conducted. The results showed that the PRV antigen was widely localized in the lymphocytes of the tonsils (Figure 5I) and mainly distributed in alveolar epithelial cells and infiltrated lymphocytes in lungs (Figure 5J), while the positive staining was mainly in the neurons of the brains (Figures 5K,L). However, no positive staining was detected in the above tissues of the control pigs (Figures 5M–O). Additionally, the analyses of viral loads in tissues also showed that the viral load of the tonsils was highest, with 1 × 10^3.16^ TCID_50_/mL, and the viral loads of the lungs and brains were similar (Figure 5P).

**Figure 6:**
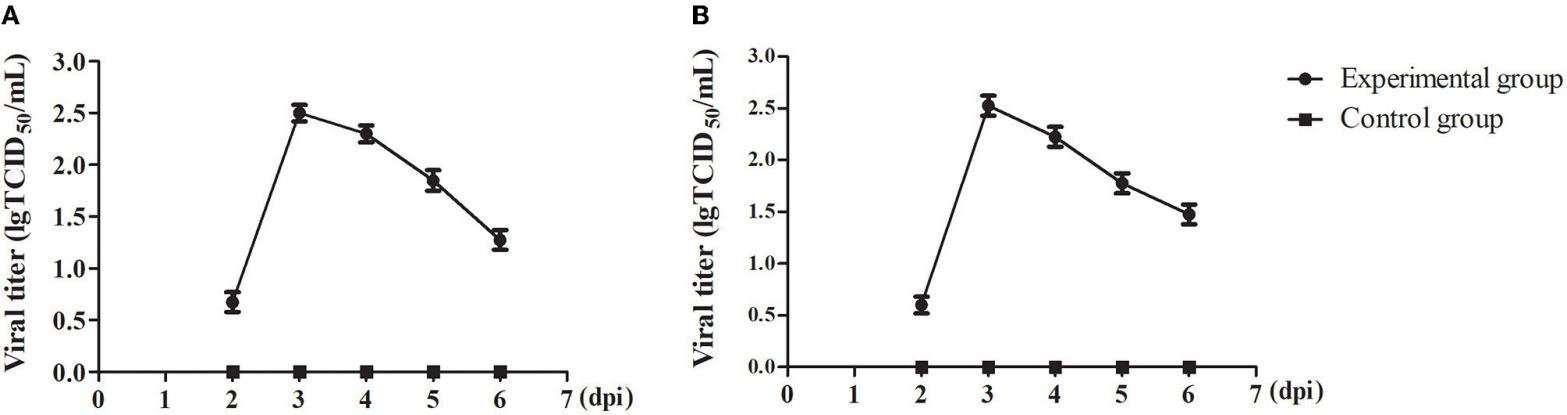
The viral shedding in swabs of the PRV-infected pigs. **(A)** TCID_50_ of PRV in oropharyngeal swabs. **(B)** TCID_50_ of PRV in rectal swabs. All data were expressed as means ± standard deviations (SD).

To understand the viral shedding patterns in pigs infected with the PRV variant strain, oropharyngeal and rectal swabs were collected from 2 to 6 dpi. The viral titer results showed that no virus was detected in the control pigs, but the infected pigs were releasing the virus via both routes as early as 2 dpi. In the oropharyngeal swabs, the titer of shed virus was 1 × 10^0.68^ TCID_50_/mL at 2 dpi, reaching a maximum value of 1 × 10^2.5^ TCID_50_/mL at 3 dpi, and then decreasing sharply at 5 dpi to about 1 × 10^1.85^ TCID_50_/mL (Figure 6A). A similar pattern of change was observed in the rectal swabs. Here, the viral titer was 1 × 10^0.6^ TCID_50_/mL at 2 dpi, reaching up to 1 × 10^2.53^ TCID_50_/mL at 3 dpi, and then decreasing to about 1 × 10^1.48^ TCID_50_/mL at 6 dpi (Figure 6B).

## Discussion

PRV has been deservedly recognized as a major infectious disease. Although efforts to eradicate this disease have been initiated and much progress has been made, a high positive rate of PRV infection still exists on swine farms. Previous studies have shown that the positive rate of PRV *gE* antibody in the swine farms of northern China has been greater than 50% (14, 15), and co-infections of PRV and other viruses were common in clinical cases, such as porcine reproductive and respiratory syndrome virus, classical swine fever virus, and porcine circovirus type 2 (16). In the current study, a large number of serum samples were collected from 2018 to 2020 in Shandong Province, China. The detection results showed that the positive rates of PRV *gE* antibody were at a high level, with 38.20% in 2018. But this dropped to 18.12% in 2020, displaying an apparent declining trend in this investigation compared to data from 2015 to 2017 in Shandong Province (16). Recently, a retrospective analysis of 256,326 serum samples from 2011 to 2021 in China found that 76,553 specimens manifested positive for PRV *gE* antibody, with an average 29.87% PRV positive rate in pigs (2). The seroprevalence rate in Jiangsu, Henan, and Jiangxi from 2018 to 2019 was over 35% (17). This indicated that the seroprevalence rate of PR in Shandong was similar to that in the whole country before, but it had showed a declining trend recently, which may be due to enhanced biosafety measures. The intensive pig farms are paying more attention to biosafety than ever before, which may be beneficial for the prevention and control of PRV infection. However, it was interesting that the positive rate of *gE* antibody in sows has always been high in recent years (6, 15), indicating that sows have faced the greatest risk of PRV infection. Because PRV can spread via vertical transmission, so infected piglets and growing–finishing pigs may induce persistent and circulatory infection in the herds, leading to high mortality and economic losses. Additionally, several risk factors associated with the occurrence of PR, such as feeding patterns, seasons, and categories of pigs should be emphasized as well (2).

From 2011 to 2021, the overall nucleic acid positive rate of PRV in pigs was 11.5% (2), and the prevalence of PRV was associated with regions (18). In the current study, a total of 97 suspected cases of PRV infection were collected to detect PRV using PCR. The detection results showed 13 samples were positive for PRV and the PRV-positive rate was 13.40%, similar to the nationwide prevalence level. The nucleotide homology analysis of the *gC, gB, gE*, and *TK* genes of four PRV strains obtained in this study showed that they shared high homology with each other and were 99% identical to the variant PRV XJ5 strain, suggesting that these four PRV strains were all variants. Actually, variant PRV strains have been spread widely in China since the outbreak in late 2011 (19). The variant XJ5 strain was isolated from diseased piglets in Jiangsu Province in 2015, and the complete genome sequence and biological characteristics of this strain had been studied (20). Due to high genetic variation of the *gC* gene, PRV strains can be divided into two genotypes based on phylogenetic analysis of the *gC* gene (4). Strains from genotype II are found almost exclusively in China (21). Genotype II can be further divided into classical and variant strains (5). The phylogenetic tree analyses of *gC, gB, gE*, and *TK* genes of four PRV strains showed that they were clustered to an independent branch and were highly homologous to previously domestic variant PRV strains, but there were significant differences compared with foreign strains, such as Kaplan and Becker. These findings were consistent with other reports (14), and it was speculated that the variant PRV strains may have evolved from the classical epidemic strains in China.

Zhou reported that the PRV variant strain XJ5 was more virulent than the classical strain Ra in 12-week-old pigs (22). To further determine the pathogenicity of variant PRV strain isolated in our study of pigs, 16-week-old pigs were inoculated with the PRV variant SD1 strain. The results showed that the five pigs all died within 1 week after the challenge and displayed severe clinical symptoms, such as hemorrhage of the tonsil mucosa with a layer of yellow-white exudate, and swelling, and necrosis of the lungs. Histopathology analysis demonstrated the typical microscopic lesions in the tonsils, brains, and lungs, including lymphocyte necrosis of the tonsils, viral encephalitis, and interstitial pneumonia. The results of immunohistochemistry staining were similar to H&E staining. There were many positive staining signals in the brain and tonsil, and high viral titers were also observed in both organs, indicating that PRV had strong neurotropic characteristics and the tonsil was an important target organ of PRV infection. PRV may be able to hide its genome from the immune system by destroying immune cells in the tonsil (23). The above findings indicated that the variant PRV strain obtained in our study also had strong pathogenicity in pigs. However, the virulence of the challenge variant strains and the age of the pigs may influence the clinical outcome (22). In addition, viral shedding in oropharyngeal and rectal swabs of PRV-infected pigs was found as early as 2 dpi in our study, suggesting that the virus could be discharged into the environment within a short time after infection, resulting in the rapid spread of the disease.

The current vaccine strain Bartha-K61 is clustered with genotype I. The early Ea and Fa vaccine strains used in China belonged to genotype II, but they were classical strains, whereas the prevalent PRV strains isolated after 2012 were almost all variants (5). Thus, these vaccines may not guarantee complete protection. Recently, Huang et al. reported that the prevalent PRV strain isolated from Sichuan was a natural recombinant strain of clade 1 and clade 2 in China (24). This finding indicated the ongoing evolution of variant PRV strains, suggesting that existing vaccines could not produce 100% protection against the variant strains (21). However, other studies have proved that pigs vaccinated with a high dose of Bartha-K61 (1 × 10^6.3^ TCID_50_/pig) were resistant to the infection of variant PRV (22). Therefore, the specific relationships between the dose of vaccine used, the virulence of the variant PRV, the immunization age of the pigs, and the immune protection will require further deep investigation.

In summary, the results of this study clearly demonstrate that variant PRV strains are still endemic at high levels in herds in Shandong Province, and there is no significant genetic variation in the epidemic strains. Pigs are highly sensitive to the variant PRV strains. Also, more attention should be paid to continuous surveillance of PRV and the development of updated vaccines.

## Data Availability Statement

The original contributions presented in the study are included in the article/supplementary material, further inquiries can be directed to the corresponding author/s.

## Ethics Statement

The animal study was reviewed and approved by the Animal Care and Use Committee of Yangzhou University.

## Author Contributions

QR performed the experiments and wrote the manuscript. HR collected a large number of samples. JG and JW detected serum samples and isolated PRV. LJ conducted animal experiments. SG designed the experiment, reviewed the manuscript, and approved the submission. All authors contributed to the article and approved the submitted version.

## Funding

This study was funded by grants from the National Key R&D Program (2016YFD0500704-2), the Novel Agricultural Research Program of Jiangsu Province (SXGC[2017] 231), the Key R&D Program of Jiangsu Province (BE2020320), the funding from the Priority Academic Program Development of Jiangsu Higher Education Institutions (PAPD), and the Science and Technology Support Program of Jiangsu Province (BE2014355) as well as the earmarked fund for Jiangsu Agricultural Industry Technology System (JATS[2018]221).

## Conflict of Interest

HR was employed by the company DSM Vitamin Co., Ltd. (Shandong). The remaining authors declare that the research was conducted in the absence of any commercial or financial relationships that could be construed as a potential conflict of interest.

## Publisher's Note

All claims expressed in this article are solely those of the authors and do not necessarily represent those of their affiliated organizations, or those of the publisher, the editors and the reviewers. Any product that may be evaluated in this article, or claim that may be made by its manufacturer, is not guaranteed or endorsed by the publisher.
